# The cellular composition and function of the bone marrow niche after allogeneic hematopoietic cell transplantation

**DOI:** 10.1038/s41409-022-01728-0

**Published:** 2022-06-11

**Authors:** Flavia Peci, Linde Dekker, Anna Pagliaro, Ruben van Boxtel, Stefan Nierkens, Mirjam Belderbos

**Affiliations:** 1 Princess Máxima Center for Pediatric Oncology, Utrecht, The Netherlands; 2 Oncode Institute, Utrecht, The Netherlands; 3 Center for Translational Immunology, University Medical Center Utrecht, Utrecht, The Netherlands

**Keywords:** Haematopoietic stem cells, Stem-cell research

## Abstract

Allogeneic hematopoietic cell transplantation (HCT) is a potentially curative therapy for patients with a variety of malignant and non-malignant diseases. Despite its life-saving potential, HCT is associated with significant morbidity and mortality. Reciprocal interactions between hematopoietic stem cells (HSCs) and their surrounding bone marrow (BM) niche regulate HSC function during homeostatic hematopoiesis as well as regeneration. However, current pre-HCT conditioning regimens, which consist of high-dose chemotherapy and/or irradiation, cause substantial short- and long-term toxicity to the BM niche. This damage may negatively affect HSC function, impair hematopoietic regeneration after HCT and predispose to HCT-related morbidity and mortality. In this review, we summarize current knowledge on the cellular composition of the human BM niche after HCT. We describe how pre-HCT conditioning affects the cell types in the niche, including endothelial cells, mesenchymal stromal cells, osteoblasts, adipocytes, and neurons. Finally, we discuss therapeutic strategies to prevent or repair conditioning-induced niche damage, which may promote hematopoietic recovery and improve HCT outcome.

## Background

Allogeneic hematopoietic cell transplantation (HCT) is a potentially curative treatment for patients suffering from hematologic malignancies, red blood cell disorders, bone marrow failure, severe immune deficiency, and certain metabolic disorders. About 20,000 and 40,000 allogeneic transplants are performed annually in Europe and the United States, respectively, and numbers are increasing [1, 2]. However, 5–10% of HCT recipients experience graft failure, which is often fatal [3]. Furthermore, poor graft function affects up to 20% of HCT recipients and predisposes to infections, viral reactivations, bleeding complications, relapsed malignancy, and overall mortality [4].

Successful HCT requires depletion of the recipient’s blood and immune system, followed by administration of donor hematopoietic stem cells (HSCs) which home and engraft the recipient’s BM and reconstitute all the blood cell lineages. Depletion of the recipient’s blood and immune system is achieved by pre-HCT conditioning, which consists of combinations of chemotherapy, radiotherapy and lymphodepleting agents. Post-transplant hematopoietic recovery typically occurs in phases: while innate immune cells and thrombocytes generally recover within weeks after HCT, complete reconstitution of adaptive immunity can take months to even years [5]. The slow reconstitution of the adaptive immune system is a result of ineffective thymic recovery, due to damage to the thymus by pre-HCT conditioning. Of note, T cells reconstitution consists in two phases: first, homeostatic proliferation of T cells from the graft; second, recovery of the thymus and thymic output. Although anti-Thymocyte Globulin (ATG) treatment can affect both stages, the homeostatic T cell proliferation is mainly impacted. Moreover, aGvHD and cGvHD can decrease thymic output, as reviewed by Velardi et al. (2021) [6]. Overall, the dynamics of post-transplant hematopoietic and immune reconstitution is one of the most important determinants of HCT-related complications and survival [7].

Host HSCs, as well as transplanted HSCs, require support of a specialized bone marrow (BM) microenvironment, known as the “niche”. The concept of a niche was first introduced in 1978 by Schofield, who postulated that the fate of a stem cell is dictated by the environment in which it resides [8]. Reciprocal interactions between HSCs and their niche regulate HSC quiescence, self-renewal, proliferation, differentiation, mobilization, and homing [9]. Conditioning-induced niche damage also involves non-hematopoietic cells [10, 11], which may further affect hematopoietic recovery after HCT [9, 12] and predispose to prolonged cytopenia, HSC non-engraftment, and poor graft function [11, 13].

Recent developments in single-cell sequencing and imaging have greatly improved our understanding of the cellular composition of the BM niche [14, 15]. These studies uncovered a great level of complexity in the cellular and molecular constituents of the BM niche, as well as in the mechanisms by which they regulate HSC behavior. However, thus far, most of these studies have been performed in mice, and studies in humans are only beginning to appear.

Here, we review current knowledge on the BM niche in the context of HCT. We summarize the effects of pre-HCT conditioning on each of the distinct BM niche cell types and on the mechanisms by which they support post-HCT hematopoietic regeneration. Furthermore, we discuss strategies to prevent or treat conditioning-induced niche damage, which may ultimately contribute to improved HCT outcome.

## Architecture of the bone marrow niche

**Fig. 1:**
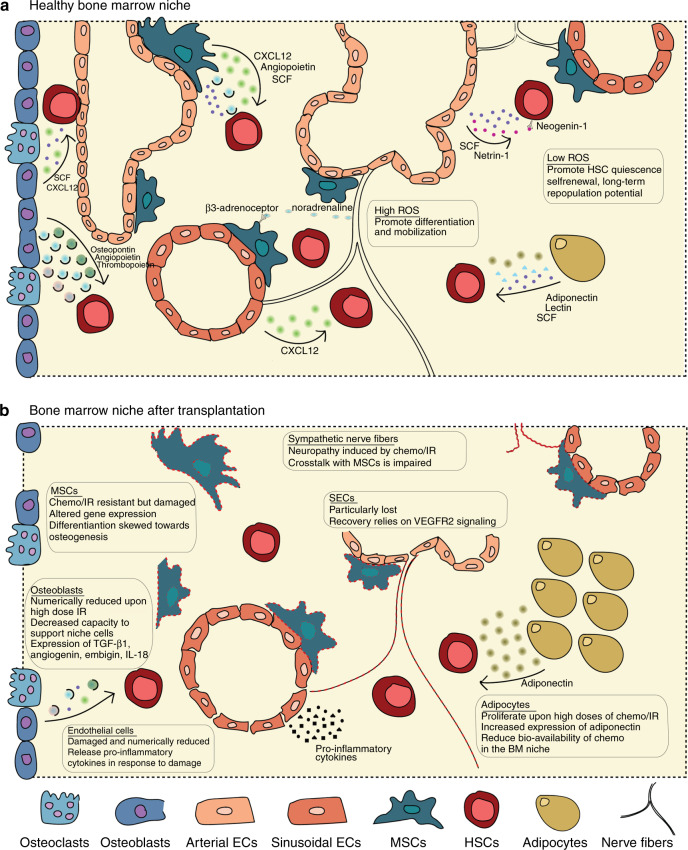
Composition and function of the bone marrow niche after HCT. Schematic overview of the healthy bone marrow niche (**a**) and the bone marrow niche after hematopoietic cell transplantation (**b**). SCF Stem Cell Factor, CXCL12 C-X-C Motif Chemokine Ligand 12. VEGFR2 Vascular Growth Factor Receptor 1, TGF-β1 Transforming Growth Factor beta 1, ROS Reactive Oxygen Species, MSCs Mesenchymal Stromal Cells, ECs Endothelial Cells, HSCs Hematopoietic Stem Cells, IR irradiation.

BM is a cell-dense, semi-solid tissue localized in the central (or medullary) cavities of axial and long bones. The BM is highly vascularized by an abundant heterogeneous network of blood vessels, which serves to supply nutrients, oxygen, and signaling molecules, while removing waste products. Nevertheless, BM is a relatively hypoxic microenvironment [16], critically regulating HSC metabolism and quiescence; high reactive oxygen species (ROS) levels promote HSC differentiation and mobilization, whereas low levels of ROS promote HSC quiescence, self-renewal, and long-term repopulating potential [17, 18]. The BM niche vasculature is supported by an extensive network of multipotent mesenchymal stromal cells (MSCs), which can give rise to osteoblasts, chondrocytes, and adipocytes [19, 20]. In addition, bone as well as BM are highly innervated by autonomic and nociceptive nerve fibers and associated Schwann cells [21]. Below, we will discuss how these BM niche populations may be influenced by transplantation procedure and the subsequent effect on hematopoietic recovery after HCT Fig. 1.

## Niche cells and their impact on hematopoietic recovery after HCT

### Endothelial cells

Endothelial cells (ECs) form a monolayer that constitutes the inner lining of blood vessels and facilitate blood flow, enable exchange of nutrients and waste products, and regulate vascular tone and blood coagulation. Based on their localization within the BM vasculature, ECs can be classified as arteriolar endothelial cells (AECs) or sinusoid endothelial cells (SECs) [22] that differ in signaling molecules and modulation of the microenvironment, thus establishing distinct vascular niches that can instruct HSCs [22, 23]. AECs are part of arteriolar vessels with low plasma penetration and maintain a relatively hypoxic environment [22, 24]. They are a major source of netrin-1, which, through interaction with its receptor neogenin-1, serves to maintain HSC quiescence and self-renewal [25]. Finally, AECs are the predominant secretors of EC-derived stem cell factor (SCF) in the BM [26]. Conversely, SECs are part of more permeable sinusoidal vessels, resulting in high plasma penetration and exposure of perivascular HSCs to higher levels of ROS [22, 27]. They express high levels of C-X-C motif chemokine ligand 12 (CXCL12), required for stem cell homing [28]. Altogether, these data show that AECs are thought to support more primitive, quiescent HSCs, whereas SECs support HSC proliferation and mobilization [22].

Increasing evidence indicates that ECs play an important role in hematopoietic recovery after HCT, by production of several hematopoietic stem and progenitor cell (HSPC)-supporting molecules. In mice, engraftment of transplanted HSPCs after either 5-fluorouracil (5-FU) or irradiation depends on recovery of SECs, which is mediated through activation of vascular endothelial growth factor receptor 2 (VEGFR2) signaling [29]. Inhibition of this signaling, through conditional deletion of VEGFR2, results in disorganized regeneration of SECs, delayed hematopoietic recovery and persistent life-threatening pancytopenia [29]. Furthermore, EC-specific expression of Tie2 [30], Jagged-1 [31], and Jagged-2 [32] have all been shown to support hematopoietic regeneration after myeloablative injury, by promoting regeneration of the vascular niche (Tie2) or by activating Notch signaling in HSPCs (Jagged-1 and Jagged-2). Finally, a subtype of capillary ECs expressing *Apelin* (Apln^+^ ECs), increases substantially after irradiation and is critical for post-transplant hematopoietic recovery in mice [33]. Interestingly, elimination of HSPCs by diphtheria toxin phenocopied the vascular changes observed after irradiation or 5-FU, indicating that HSCs actively maintain their niche, and vice versa [33].

In humans, recent studies identified a subset of BM ECs, CD105 (*endoglin*)-expressing ECs, which are nearly absent during homeostatic hematopoiesis but are enriched in fetal BM and during regeneration upon chemotherapeutic injury [24]. These ECs express high levels of interleukin-33 (IL-33), which promotes the expansion of both hematopoietic precursor cells and other EC subsets ex vivo [24]. Interestingly, a subset of these cells, CD105^+^CD271^+^ ECs, co-express endothelial as well as stromal markers and have the potential to convert to stromal progenitor cells and their downstream progeny [24]. Upon subcutaneous implantation in mice, these human CD105^+^CD271^+^ EC-derived cells formed pellets consisting of human bone, cartilage, adipocytes and blood vessels which recruited hematopoietic cells, supporting their in vivo niche-regenerative capacity [23].

In the context of HCT, ECs are exposed to a variety of damaging stimuli. In mice, irradiation or administration of 5-FU causes loss of ECs in a dose-dependent manner [29]. In human patients, conditioning with high-dose cyclophosphamide or busulfan is associated with increased risk of EC-related disorders, such as veno-occlusive disease/sinusoidal obstruction syndrome, thrombotic microangiopathy, capillary leak syndrome and idiopathic pneumonia syndrome [34]. Furthermore, bacterial endotoxins, inflammatory cytokines and calcineurin inhibitors have all been associated with EC injury [34], which could in turn impair hematopoietic recovery.

ECs may provide an opportunity to promote hematopoietic recovery after HCT, by co-infusion of healthy ECs with the stem cell product, or by protecting these cells from conditioning-induced damage. In mice, co-infusion of ECs together with hematopoietic cells improves HSC repopulating activity, engraftment, and survival after irradiation, compared to infusion of hematopoietic cells only [35]. The beneficial effect of EC co-infusion on hematopoietic recovery is even more prominent when the ECs are pre-treated with the Wnt-antagonist Dickkopf1 (Dkk1), which induces secretion of several proteins known to promote hematopoietic regeneration, including granulocyte colony-stimulating factor (G-CSF) and VEGF [36]. Strategies to protect recipient ECs from chemotherapy-induced damage include administration of pigment endothelial derived factor (PEDF) [37], defibrotide [38], and N-acetyl-L-cysteine (NAC) [39]. Although PEDF and defibrotide have been shown to improve hematopoietic recovery in mice, their effects in humans are still unknown. Prophylactic oral NAC treatment was shown to be safe and effective in preventing poor hematopoietic reconstitution in human HCT-recipients, suggested to be a result of improved BM EC function [40]. A Phase III, open-labeled, randomized clinical trials is currently recruiting to further investigate NAC for prevention of poor hematopoietic reconstitution in patients receiving an HCT (Trial no. NCT03967665).

### Mesenchymal stromal cells

Mesenchymal stromal cells (MSCs) are a rare (~0.001–0.01%) component of the BM niche. MSCs were first described in 1968 as a population of adherent cells of the BM, which exhibited a fibroblast-like morphology and which can differentiate in vitro into bone, cartilage, adipose tissue, tendon, and muscle [40]. BM MSCs co-localize closely with HSCs and regulate HSC homeostasis through the production of soluble factors, including CXCL12, angiopoietin, and SCF, which are key factors for HSC maintenance. In recent years, advances in flow cytometry and cell-tracing methods have identified multiple MSC subsets, with distinct impact on HSC behavior. CD271^+^ and CD271^+^/CD146^−/low^ MSCs are bone-lining cells that support long-term, quiescent HSCs in areas with low oxygen tension. In contrast, CD271^+^/CD146^+^ MSCs are located in the perivascular region where they support more proliferative HSCs [41]. In mice, a specific type of perivascular MSCs, Nestin^+^/NG2^+^ MSCs, produces high levels of CXCL12 and angiopoietin [20, 42]. Depletion of these cells using Nestin-Cre results in loss of HSCs, supporting the HSC-supporting role of these cells in the murine BM niche.

MSCs are extensively studied in the context of HCT. In the majority of HCT recipients, MSCs remain of recipient origin, indicating that these cells are not fully eradicated by myeloablative conditioning [43, 44]. The mechanisms that allow MSCs to survive pre-HCT conditioning regimens that are lethal to hematopoietic cells remain incompletely understood, and may involve more efficient recognition of DNA damage, double strand break repair and evasion of apoptosis [45, 46]. Conversely, one might hypothesize that MSCs can simply tolerate a higher mutational load than hematopoietic cells, for instance, by expressing translesion synthesis polymerases [47]. Although recipient MSCs may remain relatively viable after conditioning, they do accumulate damage [45]. For example, in vitro irradiation of human MSCs results in accumulation of DNA double-strand breaks [48], altered gene expression [49], skewed differentiation towards osteogenesis [49], and induction of senescence [50]. Interestingly, reports have shown that in recipients transplanted with BM and PB grafts, part of the MSC pool after HCT was of donor-origin [51, 52]. Therefore, it will be of interest to investigate how donor-recipient MSC chimerism and conditioning-induced damage relate to post-HCT hematopoietic function.

Because of their regenerative and immune-regulatory properties, MSCs are used as a clinical therapy for a variety of degenerative and inflammatory diseases, including articular cartilage defects, cardiac diseases, inflammatory bowel disease, and severe COVID-19 [53]. In the context of HCT, MSCs have been used to enhance HSC engraftment and to treat steroid-resistant aGvHD. The use of MSCs for aGvHD is beyond the scope of this review and has been reviewed elsewhere [54]. In phase I/II trials in human allo-HCT recipients, co-infusion of MSCs together with hematopoietic cells was safe and resulted in prompt engraftment in 144 out of 146 recipients [54], compared to 5–10% risk of graft failure in historic controls. Whether this apparent improvement is due to niche-restoring or immunosuppressive effects remains to be defined. Thus far, no comparative phase III studies have studied the role of MSC infusions in the prevention or treatment of non-engraftment after HCT. The feasibility of such studies is hampered by the rarity of graft rejection, the heterogeneity of the patient group and of the MSC cell product, thus requiring large numbers of patients. To facilitate such studies, it will be of interest to investigate the niche prior to HCT, to identify potential biomarkers of increased niche damage in HCT recipients, who are most likely to benefit from niche-correcting strategies.

### Osteolineage cells

Osteolineage cells are a heterogeneous pool of bone-forming cells of various developmental stages, including pre-osteoblasts, osteoblasts, and terminally differentiated osteocytes [55]. Osteolineage cells were among the first niche cell types to be implicated in the regulation of HSCs [56]. Early mouse studies showed that long-term repopulating (LT-)HSCs co-localize closely with osteoblasts. Osteoblasts secrete several factors required for HSC maintenance, such as CXCL12 [57], SCF [57], angiopoietin [58], thrombopoietin [59], and osteopontin (OPN) [57]. Finally, the number of osteoblasts in the niche is closely correlated with the number of HSCs [57, 60], and conditional ablation of osteoblasts results in loss of lymphoid, erythroid, and myeloid hematopoietic progenitor cells from the BM [61].

However, more recently, the role of osteolineage cells in HSC regulation has been subject of debate. For instance, whereas osteoblasts produce HSC-supporting molecules, they may not be the predominant source of these factors. Hepatocytes, and not BM cells, are likely the major source of thrombopoietin [62] and HSCs and stromal cells are the main producers of BM angiopoietin [63]. In addition, selective deletion of *CXCL12* or *SCF* from murine osteoblasts has little effect on HSCs [28, 64]. Furthermore, recent 3D imaging studies in mice have shown that the majority of endogenous HSCs lie adjacent to BM blood vessels, in close association with endothelial and mesenchymal cells, and that only a minority of HSCs is localized in direct contact with BM osteoblasts [19, 65]. In summary, these studies suggest that osteolineage cells may be less important for HSC maintenance during homeostatic hematopoiesis than previously thought. Notably, osteolineage cells have been shown to regulate more committed hematopoietic progenitor cells in mice [57, 63, 64], and their potential role during hematopoietic regeneration, in mice as well as in humans, remains to be defined.

Conditioning-induced damage to osteolineage cells is thought to underlie bone-related complications after allo-HCT, including bone loss, osteopenia, osteoporosis, and avascular necrosis of bone [66]. In vitro chemotherapeutic treatment of murine [67] as well as human [68] osteoblasts with VP16 or melphalan resulted in decreased production of CXCL12 and reduced capacity to support immature B progenitor cells and CD34 + BM cells [68]. Similarly, irradiation induces several functional defects in osteoblasts, such as decreased production of extracellular matrix components [69], impaired proliferation [69] and induction of apoptosis [70]. Notably, in addition to pre-HCT conditioning, various other HCT-related exposures may compromise osteoblast numbers and/or function after HCT, including corticosteroids [71], calcineurin inhibitors [66], nutritional deficiencies and G-CSF [72].

Several studies have attempted to prevent and/or restore conditioning-induced damage to osteolineage cells, to prevent bone complications after HCT and/or to accelerate hematopoietic recovery. Strategies for prevention and treatment of bone loss are excellently reviewed by McCune et al. [66]. In mice, parathyroid hormone (PTH) injection increases the number of osteoblasts and HSCs in the BM, and improves post-HCT survival [60]. However, a subsequent phase II study in human HCT recipients was halted early because of excessive treatment-related mortality [73, 74]. In the 13 evaluable patients, no beneficial effect of PTH on hematopoietic engraftment was observed [73, 74], again suggesting that the impact of osteolineage cells on hematopoietic recovery may be less evident than previously thought.

### Adipocytes

Bone marrow adipocytes (BMAs) differentiate from MSCs and comprise a heterogeneous population of cells. Although BMAs were initially considered simple “fillers” of marrow space, increasing evidence indicates that they actively contribute to hematopoiesis [75, 76]. BMAs produce adiponectin, which stimulates HSC proliferation in vitro [77]. During ageing, the number of adipocytes in the BM niche increases progressively, gradually replacing sites with hematopoietic activity [57, 78]. Furthermore, in mice, adipocyte content differs between different bones and is negatively correlated with HSPC content [78].

In mice [11] as well as in humans [79], chemotherapy and irradiation are associated with increased BM adipocyte content, potentially contributing to (transient) hematopoietic aplasia. Depletion of BM adipocytes, either by genetic engineering (fat-free A-ZIP/F1 mice) or by treatment with the PPARγ inhibitor Bisphenol-A-DiGlycidyl-Ether, resulted in accelerated hematopoietic recovery after irradiation [78, 80]. Conversely, BMAs have also been reported to promote hematopoietic regeneration. For instance, adiponectin-null mice showed delayed hematopoietic recovery upon myeloablative injury compared to wild type mice [81]. Furthermore, murine BMAs produce SCF, and adipocyte-specific deletion of SCF inhibited hematopoietic regeneration after irradiation or chemotherapy, resulting in increased transplant-related mortality [75]. Interestingly, treatment of murine HCT recipients with simvastatin, a drug already used in the treatment of hypercholesterolemia, prevents radiotherapy-induced BM adipogenesis and improves HSC engraftment [82]. Taken together, the impact of BMAs on steady-state hematopoiesis and hematopoietic regeneration remains controversial and requires future studies, particularly in humans.

### Nerve fibers

BM nerves regulate the proliferation, differentiation, and migration between the BM and extramedullary sites of HSPCs, during homeostatic hematopoiesis and after HCT. Most studies have focused on the sympathetic nervous system (SNS) [20, 85], although more recently, a role for the parasympathetic nervous system was also proposed [83]. Sympathetic nerve fibers release noradrenalin, which facilitates HSPC egression from the BM towards extramedullary sites [21]. In fact, circadian changes in the balance between sympathetic and parasympathetic signaling are thought to underlie the daily oscillations in HSC proliferation and migration, as reviewed by Mendez-Ferrer et al. (2009) [84]. The interaction between the SNS and HSCs is (at least in part) mediated via niche cells, as binding of noradrenalin to the β_3_ adrenergic receptor expressed by stromal cells results in downregulation of CXCL12, the key niche retention chemokine [85]. In addition, noradrenalin-mediated activation of the β_3-_adrenergic receptor on HSPCs promotes HPSC mobility and proliferation [86]. The importance of sympathetic nerve signaling for HSC maintenance is exemplified by recent murine studies, demonstrating that loss of sympathetic nerves or β_3_ adrenergic signaling in the BM results in premature HSC ageing, which can be rescued by supplementation of sympathomimetic agents [87].

Chemotherapy and/or irradiation can be particularly neurotoxic, inducing transient or persistent sympathetic neuropathy which may contribute to hematopoietic dysfunction. In mice, chemotherapy with cisplatin or 5-FU is associated with decreased numbers of SNS fibers in the BM [10]. In humans, many cancer survivors suffer from radiation-induced neuropathy [88]. Similarly, several chemotherapeutic drugs (e.g., vinca alkaloids, taxanes, platinum-based agents) and calcineurin inhibitors commonly induce severe peripheral neuropathy [89, 90].

Whether and how chemo- or radiotherapy-induced neuropathy impacts on post-transplant hematopoietic regeneration remains incompletely understood. In mice, cisplatin-induced sensory neuropathy is associated with impaired bone marrow regeneration and decreased survival after HCT [10]. In these mice, selective depletion of adrenergic innervation in the BM by 6-hydroxydopamine resulted in prolonged BM aplasia, both after chemotherapeutic myeloablation as well as after irradiation [10]. This effect was specific to neurons, because protection from chemotherapy-induced nerve damage by deletion of *Trp53* in sympathetic neurons, or by administration of neurotrophic compounds, could restore hematopoietic recovery [10]. Furthermore, administration of hematopoietic growth factors, such as G-CSF and granulocyte-macrophage colony-stimulating factor (GM-CSF) is associated with increased expression of neuronal receptors on HSPCs, enhancing their proliferation and repopulation capacity [86]. Importantly, although sympathetic neurons are important regulators of HSCs, they also impact on the behavior of other niche cell types, for example MSCs, thereby indirectly influencing hematopoietic cells [85, 91].

Beyond the SNS, the nociceptive nervous system has also been shown to impacts on HSC homing and migration [92]. Treatment of mice with calcitonin gene-related peptide (CGRP), the main nociceptive neurotransmitter, substantially increased G-CSF induced HSC mobilization into the peripheral blood, at the expense of BM HSC content [92]. CGRP interacts directly with HSCs, via receptor activity modifying protein 1 (RAMP1) and the calcitonin receptor-like receptor (CALCRL), increasing intracellular cAMP levels which facilitate HSC mobilization. Intriguingly, mice fed capsaicin-containing food, a known nociceptive activator, also displayed significantly enhanced HSC mobilization. As HCT is associated with many painful stimuli and analgesic medications, it will be of interest to investigate the impact of nociceptive signaling on hematopoietic recovery in this context.

## Conclusions

Recent technological advances have allowed deconstruction of the BM niche and provide insight into the mechanisms by which the niche is affected by HCT conditioning and how it regulates HSC behavior, during homeostatic hematopoiesis and hematopoietic regeneration. HCT is associated with multiple changes in the BM niche, including dysfunction of ECs and neurons, accumulation of DNA damage in MSCs, reduced numbers osteoprogenitor cells and increased numbers of adipocytes, which may collectively impair hematopoietic reconstitution (Table 1). To improve HCT outcome, several niche-directed strategies have been explored, including antibody-based conditioning [93, 94], infusion of extracellular vesicles derived from BM-MSCs [95, 96], co-infusion of HSCs with autologous or allogeneic MSCs or ECs [35, 97], inhibition of adipogenesis by simvastatin treatment [82], or the use of endothelial [37–39], and neuroprotective compounds [10] (Table 2).

**Table 1:** Causes and consequences of pre-HCT conditioning on the BM niche.

Cell type	Factors produced	Role in the normal HSC niche	Impact of HCT conditioning	Consequences on hematopoietic recovery	Model system	Ref.
Endothelial cells	CXCL12, SCF, Angiopoietin	Maintenance of quiescent HSCs (AECs), HSC migration and proliferation(SECs)	Loss of ECs in a IR dose-dependent manner; increased risk of EC-related disorders in human	Engraftment depends on recovery of SECs through activation of VEGFR2; signaling inhibition results in delayed hematopoietic recovery	Mice, human	[29, 34]
Mesenchymal stromal cells	CXCL12, SCF, Angiopoietin	HSC homeostasis	MSCs are not fully eradicated but do accumulate DNA damage	Unclear, hematopoietic recovery might be delayed.	Human, in vitro	[45, 48–50]
Osteolineage cells	CXCL12, SCF, Angiopoietin, Thrombopoietin, Osteopontin	Regulation of more committed hematopoietic progenitor cells; HSC maintenance by osteoblasts is subject of debate.	Bone-related complications; compromised osteoblast number and function.	Unclear. The supportive effect of osteolineage cells on HSCs may be less evident than previously thought.	Mice and human (in vitro)	[66–74, 76]
Adipocytes	Adiponectin, Lectin, SCF	HSC proliferation	Increased BM adipocyte content	Controversial. Potentially contributing to (transient) hematopoietic aplasia. However, others report on their promotive role in hematopoietic regeneration.	Mice and human (in vitro)	[77, 81]
Sympathetic neurons	Noradrenalin	HSPCs proliferation, differentiation, and migration	Transient or persistent sympathetic neuropathy; loss of sympathetic fibers	Impaired bone marrow regeneration	Mice	[10]
Nociceptive neurons	CGRP	HSC homing and migration	Unknown	Unknown	NA	NA

**Table 2:** Niche-directed therapeutic strategies.

Niche cell type	Therapeutic strategy	Model system	Effect	Ref.
Endothelial cells	Co-infusion of ECs with hematopoietic stem cells; Administration of PEDF, defibrotide, and NAC.	Mice and humans (phase I/II and III)	Improved HSC repopulating activity, engraftment, and survival after irradiation; Protection of recipient ECs from chemotherapy-induced damage; Prophylactic oral NAC was safe and effective in preventing poor hematopoietic reconstitution by improving BM EC function in allo-HCT recipients; Effect will further be identified in phase III clinical trial.	[35–39] Trial no. NCT03967665 (currently recruiting)
Mesenchymal stromal cells	Co-infusion of MSCs with hematopoietic cells	Humans (phase I/II clinical trials)	Prompt engraftment of donor HSCs	Trials are reviewed in ref. [53], Trial no. NCT04247945 (currently recruiting)
Osteolineage cells	Parathyroid hormone (PTH) injection	Mice and Humans (halted at phase II)	Increases the number of osteoblasts and HSCs in the BM and improves post-HCT survival in mice. No beneficial effect on hematopoietic engraftment was observed in human HCT recipients.	[73]
Adipocytes	Simvastatin treatment	Mice	Prevents radiotherapy-induced BM adipogenesis and improves HSC engraftment	[82]
Sympathetic nervous system	Administration of hematopoietic growth factors, such as G-CSF and GM-CSF; Neuroprotection by administration of 4-methylcatechol	Mice	Increased expression of neuronal receptors on HSPCs, enhancing their proliferation and repopulation capacity; Accelerates BM regeneration	[86]
Nociceptive neurons	Unknown	NA	NA	NA

Importantly, as interplay between several functionally intact niche cell types is likely required for adequate HSC support, combination therapies may be required. Future studies are needed to compare the impact of different pre-HCT conditioning regimens on the BM niche in mice as well as in humans, to identify the BM niche cell types most susceptible to conditioning-induced damage, to assess the impact of this damage on HSC engraftment and long-term function, and to select the most appropriate treatment.
